# Genetic and environmental contributions to prosocial behaviour in 2- to 9-year-old South Korean twins

**DOI:** 10.1098/rsbl.2007.0365

**Published:** 2007-08-28

**Authors:** Yoon-Mi Hur, J. Philippe Rushton

**Affiliations:** 1 Chonnam National UniversityKwangju, South Korea; 2 University of Western OntarioLondon, Ontario, Canada N6A 5B8

**Keywords:** prosocial behaviour, twins, genetics, environment, cross-culture

## Abstract

Although over 50 twin and adoption studies have been performed on the genetic architecture of antisocial behaviour, far fewer studies have investigated prosocial behaviour, and none have done so on a non-western population. The present study examined mothers' ratings of prosocial behaviour in 514 pairs of 2- to 9-year-old South Korean monozygotic and dizygotic twins. Correlational analyses showed a tendency of increasing genetic effects and decreasing shared environmental effects with age although shared family environment effects and the moderating effects of age did not attain statistical significance in model-fitting analyses. The best-fitting model indicated that 55% (95% CI: 45–64%) of the variance in the 2- to 9-year-olds' prosocial behaviour was due to genetic factors and 45% (95% CI: 36–55%) was due to non-shared environmental factors. It is concluded that genetic and environmental influences on prosocial behaviour in young South Koreans are mostly similar to those in western samples.

## 1. Introduction

Empathy, altruism and prosocial behaviour are considered vital for the good functioning of society. Although psychological theories emphasize the importance of cognition and socialization, genes also have a role to play. Monozygotic (MZ) twin pairs share 100% of their genes, whereas dizygotic (DZ) twin pairs share 50%; thus the comparison of MZ and DZ twin similarities and differences allows for estimates to be made of genetic influences (Plomin *et al.* 2001). Several studies have found that by adulthood, approximately 50% of the variance in altruism, empathy and social responsibility is due to genes and 50% to non-genetic factors (Rushton *et al.* 1986; Rushton 2004). Over 50 twin and adoption studies have found similar results for antisocial behaviour (Rhee & Waldman 2002).

A striking result from twin and adoption studies is that, by adolescence, the 50% non-genetic variance between siblings is idiosyncratic; coming mainly from non-shared environmental sources rather than from those that are shared (Plomin *et al.* 2001; Bouchard & McGue 2003; Harris 2006). The *shared environment* (also called *common* or *between-family environment*) includes all those variables that children reared in the same family have in common (e.g. parental socio-economic status and child-rearing style); they make children growing up in the same family similar to one another. The *non-shared environment* (also called *specific* or *within-family environment*) includes all those variables that are unique to each child (e.g. an illness or chance friendship that happens to one sibling and not the other); they make children growing up in the same family different from one another.

The relative strengths of genetic and shared family effects on prosocial behaviour tend to reverse with increasing age. For example, Scourfield *et al.* (2004) studied 619 pairs of 5- to 16-year-old twins using parent and teacher reports and found that genetic effects increased from 46% in 5- to 10-year-olds to 87% in 11- to 16-year-olds, while shared environment effects decreased from 30 to 0%. Similarly, Knafo & Plomin (2006) studied 9424 twin pairs rated by their parents at the ages of 2, 3, 4 and 7 years, and by their teachers at 7 years, and found that genetic effects increased from 32% at 2 years to 61% at 7 years, while shared family effects decreased from 47 to 3%.

The great majority of twin studies have been carried out in western countries, so it remains uncertain how generalizable the ‘genetic and environmental architecture’ of prosocial behaviour is to non-western populations. The present study explores the issue with measures of prosocial behaviour in young South Korean children. To our knowledge, this is the first twin study of prosocial behaviour in a non-western population.

## 2. Material and methods

The present sample was drawn from the South Korean Twin Registry (Hur *et al.* 2006). As part of a scheduled telephone interview, the Korean version of the Prosocial Scale of the Strengths and Difficulties Questionnaire (Goodman 1997) was given to the mothers of 2- to 9-year-old twins (mean: 4.4 years; s.d.: 2.1). Zygosity was determined from chorion type in prenatal records and mothers' responses to questionnaires about physical similarity (Ooki *et al.* 1993). Only those pairs with unambiguous zygosity were included. The final sample comprised 514 twin pairs with 82 male monozygotic twin pairs (MZM), 56 female monozygotic pairs (MZF), 88 male dizygotic pairs (DZM), 103 female dizygotic pairs (DZF) and 185 opposite-sex pairs (OSDZ). The higher number of DZ than MZ pairs is due to the number of assisted pregnancies (Hur & Kwon 2005).

The Korean version of the Prosocial Scale has been found to be reliable and valid (Ahn *et al.* 2003). Mothers rated each of their children on five items regarding sharing, helping and being kind on a 3-point scale from ‘not true (0)’ to ‘certainly true (2)’. The ratings were summed over the five items to obtain a total score. The total score was approximately normally distributed (skewness: −0.12).

## 3. Results

The internal consistency reliability estimate (Cronbach's alpha) of the five prosocial items was 0.66. Age and sex effects were found. Girls had higher scores than boys (mean: 6.7 versus 6.0; s.d.: 2.2; *p*<0.01). There was no main effect for age but there was an age×sex interaction such that older girls were rated as more prosocial. The age-adjusted intraclass twin correlations in total sample were 0.56 for MZM, 0.70 for MZF, 0.19 for DZM, 0.26 for DZF and 0.27 for OSDZ twins. The higher MZ than DZ twin correlations indicated substantial genetic influences on prosocial behaviour in both males and females. Table 1 shows sex-adjusted intraclass correlations broken down by age and zygosity. The tendency for the DZ twin correlations to decrease with age while the MZ correlations remained high suggests that, while genetic effects may have increased with age, the common environmental effects decreased.

Although the results can be gleaned from the correlational analyses, we also carried out model fitting to yield parameter estimates and compare alternative models (Plomin *et al.* 2001). We used a general sex-limitation model incorporating age as a moderator (figure 1; Neale & Cardon 1992; Purcell 2002). The full model included additive genetic (A) and shared (C) and non-shared (E) environmental factors that were allowed to differ as a function of sex and age (M). The extent to which age moderates A, C and E was represented by *β*_a,_ *β*_c_ and *β*_e,_ respectively. The main effects of age were also included in the model for males (*μ*+*β*_m_M) and females (*μ*+*β*_f_M). On the basis of the degree of genetic relatedness, the correlations for A were set at 1.0 for MZ twins, at 0.50 for same-sex DZ (SSDZ) twins and at less than 0.50 for OSDZ twins. The correlation for C was set at 1.0 for all types of twins because they were raised together. The correlation for E was set at 0.0 because this variance is unique to each member of a twin pair.

The model-fitting analyses used the raw data option in Mx (Neale *et al.* 2003). Mx calculates twice the negative log likelihood (−2LL) of the data, with a chi-square difference test to evaluate between models. First, the full model was tested against a baseline where variances of the first- and the second-born MZ and DZ twins were allowed to differ. The chi-square between the baseline and full model was not significant (Δ*Χ*_68_^2^=26.6, *p*>0.99), leading to the acceptance of the full model. The full model showed that 42% of the variance was due to genetics, 17% due to shared environmental effects and 41% due to non-shared environmental effects. Next, variations were made by constraining parameters from the full model to determine the best-fitting model. A significant difference in chi-square indicates that constraining the parameter caused a significant decrease in fit of the model, whereas a non-significant difference suggests that constraining the parameter is acceptable. Table 2 gives the goodness-of-fit results for the full model and its submodels. The genetic weights were the same for SSDZ and OSDZ twins (model 2), suggesting that the same set of genes are operating in males and females. To test whether or not age moderated the effects, we eliminated the age moderator from model 2. Age had no significant moderating effect on genetic and environmental influences (model 3). Age showed a significant main effect on the mean for females only (models 7 and 8). Elimination of C did not significantly worsen the fit for either sex (model 4), whereas removing A did (model 5). These results suggest that genetic factors are critical, while shared environmental factors are not significant. When the A and E parameters were constrained to be equal between the sexes, changes in chi-square were not significant (model 6), suggesting that the magnitudes of genetic and environmental influences on prosocial behaviour are similar in males and females. Taken together, these results suggest that the best fit was an AE model (model 7) with genetic and non-shared environmental effects, respectively, of 55% (95% CI: 45–64%) and 45% (95% CI: 36–55%) for both sexes. The main effect of age on the mean was significant for females such that the expected prosocial scores for females increased by approximately 0.11 s.d. with each year from 2 to 9.

## 4. Discussion

The 55% heritability and 45% non-shared environmentality of prosocial behaviour in this study of 2- to 9-year-old South Korean twins is within the range of estimates reported from western samples. There were indications of shared family environmental effects in the full model and at the early ages and of non-additive genetic (allelic interaction) effects at later ages (table 1). However, these effects were not statistically significant, perhaps due to the small sample size. The lack of a significant shared environment effect in the present South Korean study of prosocial behaviour is similar to results from other studies of psychological tests investigated using South Korean twins (Hur 2006, 2007). Also, on our measure, the mean level of prosocial behaviour was lower and the s.d. somewhat higher than in western samples (Melzer *et al.* 2000). Consistent with prior studies was our finding that girls were more prosocial than boys and that prosocial behaviour increased with age (at least in girls), and that no sex difference existed in the transaction of genetic and environmental influences. In the main, the results show the cross-cultural generality of genetic and environmental influences on prosocial behaviour.

Limitations on the present study include the use of a single measure of parental report, a limited age range, and a lack of power associated with the small sample size for complex model fitting. However, the parental ratings neither inflate the similarity of the MZ twins nor magnify the differences of the DZ twins and so overestimate genetic influence or underestimate shared environment effects because the variance within the DZ pairs was no different from that within the MZ pairs; nor were the mothers certain of their children's zygosity at the time of the interviews. In conclusion, heritable individual differences in prosocial behaviour emerge at a very early age among East Asians just as they do in western populations.

## Figures and Tables

**Figure 1:**
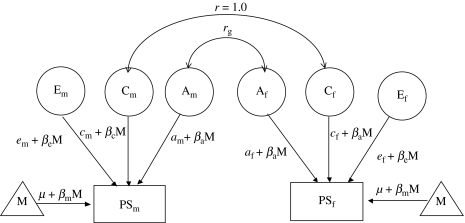
Sex-limited additive genetic (A), shared environment (C) and non-shared environment and measurement error (E) effects on the scores of the prosocial scale of opposite-sex DZ twin pairs. Subscripts m and f refer to male and female, respectively. *r*_g,_ genetic correlations; PS, prosocial scale; M, moderator (age); *β*, regression coefficient for M.

**Table 1:** Intraclass correlations for prosocial behaviour ratings on 2- to 9-year-old South Korean MZ and DZ twins. (Adjusted for sex.)

age (years)	no. of pairs	MZ twins	DZ twins
2	132	0.63	0.36
3	109	0.77	0.48
4–5	92	0.47	0.19
6	87	0.53	0.16
7–9	94	0.63	0.03
total/mean	514	0.61	0.24

**Table 2:** Model-fitting results. (A, additive genetic effects; C, shared environmental effects; E, non-shared environmental effects including measurement error; *r*_g_, genetic correlation for opposite-sex DZ twins; *β*, the main effect of age on the mean; −2LL,−2 log likelihood. Subscripts m and f refer to male and female, respectively. The best-fitting model is in italics.)

model	description	−2LL	d.f.	Δ*Χ*^2^	*p*
1	full	4375.8	1011		
2	*r*_g_=0.5	4376.3	1012	0.5	0.48
3	*r*_g_=0.5, drop all moderating effects of age	4388.0	1018	12.2	0.09
4	same as model 3, but drop C_m_ and C_f_	4388.0	1020	12.2	0.20
5	same as model 3, but drop A_m_ and A_f_	4403.8	1020	28.0	0.00
6	same as model 4, but A_m_=A_f_ and E_m_=E_f_	4393.2	1022	17.4	0.10
*7*	*same as model 6, but drop β*_m_	*4396.6*	*1023*	*20.8*	*0.06*
8	same as model 6, but drop *β*_f_	4421.1	1023	45.3	0.00
